# The Terminology of “Minor Attracted People” and the Campaign to De-stigmatize Paedophilia Originated in Pro-pedophile Advocacy

**DOI:** 10.1177/15248380251332198

**Published:** 2025-04-11

**Authors:** Michael Salter, Delanie Woodlock, Christina Farmer

**Affiliations:** 1 University of New South Wales, Sydney, NSW, Australia

**Keywords:** child maltreatment, sexual abuse, child maltreatment, offenders/perpetrators, child maltreatment

## Abstract

This note presents our response to commentary critical of our paper “A Review of Academic Use of the Term ‘Minor Attracted Persons’.” We observe that this commentary does not engage with our primary argument, conclusions, or recommendations. The authors failed to consult the supplementary material published alongside our article, failed to consider our inclusion and exclusion criteria, and spuriously accused us of underlying bias and a secret agenda. Our core criticisms of scholarship on “minor attracted people” have been left unanswered by their commentary.

The commentary to our paper contains serious allegations: that we have intentionally misrepresented the body of academic scholarship using the term “minor attracted” people (MAP), that we have sought to mislead our readers according to a secret agenda, that our scholarship is incompetent, and that we have personal hostility toward the authors we have reviewed. We will discuss and reject each of these points in turn.

From the outset, we observe the stark contrast between the outraged tone of this commentary and the actual substance of their disputes with our paper. Despite the wild scope of their accusations, the authors do not contest our central argument, conclusions, or recommendations. They do not deny that the term MAP was coined on an online forum for “boylovers,” or that MAP terminology (and the closely associated campaign to destigmatize pedophilia) has been promoted for 25 years by murky networks and organizations whose members include convicted child sex offenders and individuals who advocate for the sexual exploitation of children.

Nor do they dispute that academic scholars using the MAP term have frequently relied upon the cooperation of such networks and organizations to recruit research participants. Through their interviews and surveys, MAP researchers have documented the sympathetic self-conception of pro-pedophile networks and their self-interested agenda, without acknowledging the bias of these groups or the disastrous child protection implications of their goals. These are our core criticisms of the MAP scholarship. They are serious, and they are left untouched by the commentary.

## Intentional Misrepresentation

The authors claim that we have misrepresented MAP scholarship through selective reporting and quotations. Nonetheless, the authors do not dispute our coding of the literature, nor our conclusion based on that coding, which is that MAP scholarship is focused on the supposed shame and stigma experienced by MAP as an oppressed sexual minority.

The commenters point to examples where the sentence following a passage that we have quoted is related to that passage, which they present as evidence that we have intentionally omitted that sentence. It is obviously true that each sentence in a paragraph follows from the previous sentence and often qualifies or adds to it. It is also true that a thematic analysis involves a balance between breadth and depth. Our methodology sought to identify whether our data set (in this instance, 30 academic papers) was structured according to higher-order codes auspicing more nuanced codes. Based on our methodological approach, we see no possible conclusion from our analysis other than the one we have drawn, nor do the authors of the commentary present an alternative.

## Secret Agenda

The authors claim that our analysis is biased by a secret agenda. The second author of the paper, Salter, has been publicly critical of MAP organizations as well as academic adoption of the terminology and argumentation promoted by these organizations. The grounds for Salter’s concerns are laid out expansively in the introduction and historical overview sections of the paper. This position was not withheld from the reader but was presented transparently and at length. Nor did this position preclude new or unexpected findings that challenged prior assumptions.

We did not know what a thematic analysis of this literature would find before we embarked on it. In many respects, we were surprised by the relative coherence of the literature and the consistency of its core assumptions and logics. Nonetheless, throughout our paper, we sought to highlight examples of the diversity of opinion and perspective within MAP scholarship to avoid a one-dimensional characterization. It is interesting that the commentary authors often took exception to these examples.

## Incompetent Scholarship

The authors assert that our scholarship is incompetent on two grounds that we will address here: omission of sources and citation of media sources. The commentary contains other misreadings and straw men that are too numerous to rebut, and we assume that a capable reader who consults our paper will draw their own conclusions.

The authors claim that we have “omitted” 11 sources that were eligible for our review. This is false. They list five papers that were included in our review, as we declared in the supplementary material appended to the article. They list four papers that we excluded because they did not meet the inclusion criteria as well as a magazine article that is obviously out of scope (Sorrentino & Abramowitz, 2021). This leaves a single source (Appel, 2023) that was in scope for the review but not captured by our search, perhaps because it was not indexed by the databases we used.

The authors spuriously claim that we “centered” media sources. Our paper includes a discussion of media reactions to MAP activism and scholarship, and we cite media examples accordingly. It is somewhat ironic that they refer to a magazine article to substantiate their unfounded criticisms.

## Personal Animosity

Finally, we are curious about the authors’ assertion that we are personally hostile to them and others who work in MAP scholarship. We are aware that some MAP scholars have been the target of harassment and abuse. We condemn this and do not seek to add to it. However, MAP scholarship contains consequential, controversial, and, often, quite startling claims, among them, that pedophiles are similar in many respects to LGBTIQA+ people and that sexual interest in children should be considered normal and socially acceptable. Given their implications for LGBTIQA+ rights and child protection, these assertions should be taken seriously and subject to scholarly scrutiny. As our paper outlines, we object to these claims on empirical, practical, and political grounds, not due to personal animosity toward MAP scholars (who we assume are conducting their work in good faith, despite our differences).

However, the destigmatization campaign waged by the MAP movement and its academic allies is based on a form of special pleading that is difficult to reconcile with the existing public health and prevention scholarship but is congruent with the longer history of pro-pedophile activism from which the MAP movement emerged. We urge MAP scholars to reflect on the limitations of their current methodological approaches and underlying assumptions, and to be more transparent about the origins of MAP rhetoric and argumentation.
